# Caring for Patients’ Reproductive Healthcare During the COVID-19 Pandemic

**DOI:** 10.1177/1542305020962012

**Published:** 2020-11-23

**Authors:** Maya Bass

**Affiliations:** Department of Family, Community, and Preventive Medicine, Drexel University College of Medicine, Philadelphia, PA, USA

**Keywords:** COVID, mental health, reproductive rights, abortion, women, support

## Abstract

Abortion care requires supporting a patient through the decision to end a pregnancy, the process of having an abortion, and how to care for themselves after the decision is made. This process is nuanced in the best of times and has been exacerbated by the COVID-19 pandemic. This article provides a foundation for clergy and mental health providers on some of the issues that patients will experience accessing abortion care, specifically during the pandemic.

“Are you sure?” she asked me. Stephanie (name changed) was lying on her back, her stomach exposed and covered in ultrasound gel. I had finished establishing how far along she was in her pregnancy. I confirmed; I was sure. It was too late for her to be able to complete the abortion by taking two different medications spaced at least a day apart. Instead, she would need to have an aspiration procedure in the clinic. She started to cry. Her cloth mask absorbed her tears. I placed a gloved hand on her shoulder and helped her sit up. I reassured her. The procedure is safe. We would be in the room with her and it would be over in less than 15 minutes. We could do it today so she would not need childcare for tomorrow. She had driven over 10 hours from Texas to have the procedure done. She had known about the pregnancy for two months but due to the brief ban on abortion in Texas, her care had been postponed. She could still safely have the abortion, but it was no longer the method she wanted. Why was she still so distraught? Did I address her concerns? Often, we assume we understand, and counsel based on our own interpretation. This may mean missing the real reasons behind a patient’s tears.

Abortion care requires supporting a patient through the decision to end a pregnancy, the process of having an abortion, and how to care for themselves after the decision is made. This process is nuanced in the best of times. The effects of COVID-19 on abortion access have been drastic, but has had less to do with the virus itself, and more to do with the opposition’s willingness to use any crises to deny women, in particular poor women, their right to healthcare. This article provides a foundation for clergy and mental health providers on some of the issues that patients will experience accessing abortion care, specifically during the pandemic. Hopefully, this information will help with patient support before, during, and after an abortion.

## Deciding to End a Pregnancy

The vast majority of patients have made their decision about their pregnancy before they even contact the clinic. Abortion, like many decisions, is based on a person’s life experience, living situation, and beliefs. Patients are experts in their current situation. They know the effect of continuing a pregnancy on their spiritual, emotional, physical, and financial health. This information provides a foundation for providing comfort, reassurance, and validation to their experience. As a provider, my role is to meet the patient where every they are, honor their decision and help guide them through the safest medical option for them. *Some patients will seek guidance from clergy members or spiritual leaders in their community.* The most successful counseling of these patients is simply assessing their concerns and highlighting their values while giving what medical details are necessary for them to feel confident in their decision.

Unfortunately, many patients must also consider the politics of the state in which they live. Each state can enact laws to restrict abortion care in their states. State laws to restrict abortion, termed Targeted Restrictions against Abortion Providers (TRAP laws), have been debated in many courts. In the 2016 case *Whole Woman’s Health v. Hellerstedt*, the Supreme Court ruled that there should not be laws that put undue burden on patients seeking care. The decision was supported by an amicus brief written by many religious organizations including the Religious Coalition for Reproductive Choice, a successor organization of the Clergy Consultation Service (Dunlap, 2016). This ruling has since been used to overturn some TRAP laws and was upheld in the June 2020 decision in *June Medical Services v. Russo*. Still, many TRAP laws exist and continue to add barriers to care.

*COVID has complicated a patient’s decision.* TRAP laws made it difficult for clinics to adapt to the changes due to COVID-19. Instead of performing the majority of medical interactions using a telehealth approach, as many medical providers were encouraged to do, some states continued to require one or more in-person visits. Patients require sensitivity and reassurance that seeking this care is essential; thus, a patient’s presence in clinic is not contradicting state mandates and public health recommendations. *The uncertainty and anxiety of the pandemic have also played heavily into a person’s decision about whether to continue a pregnancy.* A common reason for getting an abortion is a lack of financial security, which many faced due to job loss and insecurity (Chae, 2017). Abortions are also used to help space desired pregnancies (Chae, 2017). In the situation of a pandemic, many patients preferred to wait to have children because of fears of accessing healthcare due to the increased risk of exposure and the inability to have support people on the labor floor or in doctor visits.

Many states require counseling prior to having an abortion. Clinics use this time to help guide patients. However, *some are required to give patients disinformation*. One example of this is that some states require that a counselor falsely claim that there is a connection between breast cancer and abortion. This has been disproven in rigorous clinical studies (National Cancer Institute (NCI), 2010). This misinformation disempowers patients as it imposes false information that may disrupt genuine decision making. A provider *or clergy member* must acknowledge the assault on a patient’s ability to provide informed consent and empower them use accurate information in their decision making.

## Deciding How to Experience an Abortion

After deciding to end a pregnancy, patients face the decision of how to have the abortion, which requires emotional and physical support, but also factual information. Patients must account for what is available in their state, the gestational age of their pregnancy (how long into the pregnancy a patient is), their current situation, and, unfortunately, how they will pay for the abortion.

In addition to the financial and emotional distress caused by COVID, there was also an increase in confusion as to where one could access an abortion. During the COVID-19 crisis, nine states temporarily banned abortion. They did this despite the fact that many medical associations including the American Medical Association (AMA), the American College of Obstetrics and Gynecology (ACOG), and the American Academy of Family Practice (AAFP) all deemed abortion essential, citing that delaying this care affects patients’ well-being and could lead to increased risks (AAFP, 2020; ACOG, 2020; AMA, 2020). Due to the tireless work of activists, non-profits, medical clinics, and these medical organizations, none of the state bans were successful in the long term. However, from March to April 2020, reproductive access was temporarily in disarray. This created another level of turmoil for patients trying to access care. Experiencing unique and unforeseen barriers can be devastating. Counselors *and clergy members* are needed to support resiliency in these patients to allow them to continue the journey to get their abortion.

Gestational limits, or how far into a pregnancy that a person can have an abortion, are, unfortunately, being determined by state legislators, not medical professionals—a fact that many patients may not recognize. Gestational limits can add to their emotional burden should they require an abortion later in pregnancy. Normalizing getting an abortion at any gestational age can alleviate the additional weight carried by these patients. It is also important to be able to support patients who may be past the state-determined gestational limit for their desired procedure. Medication abortions are now approved into the 11th week of a pregnancy; after that, the efficacy rate starts to decrease (Kapp, 2019). Thus, some patients may need to get a procedural abortion.

If, based on gestational age, a patient can choose either method, it is best to walk the patient through each process, highlighting that this decision is unique to each patient and must take into account how they may best handle the physical and emotional discomforts that come with an abortion. Guidance can highlight whether a patient prefers the comfort of their home versus the support of the clinic; childcare requirements; length of time spent passing the pregnancy; and length of time until confirmation that the process is complete. Allowing the patient to guide the conversation will typically help to identify the best method for them. Then there is the question of costs.

The cost of abortion increases with gestational age and the availability of providers in each state. It ranges from a few hundred dollars to over 1000. For the working mother (59% of women getting abortions already have at least one child) who lives paycheck to paycheck, this means going into debt or skipping meals and other essentials (Jerman, 2016). During the COVID-19 crisis this was further exacerbated as many people lost their jobs and some states temporarily closed abortion clinics, requiring patients to travel to adjacent states, further increasing costs and time away from work and family. Delays in care to acquire necessary funds are common. This can lead to frustration and hopelessness in patients who feel that they will be unsuccessful in getting enough funding. Significant delays mean that a patient may be too far along to get the type of abortion that they prefer or, unfortunately, if the delay brings them past their state’s gestational limit, the patient may not be able to access abortion in that state at all.

Some patients, due to lack of access and cost, will end up managing their own abortions. Patients will get medications from online websites to allow them to have an abortion at home. Some hotlines have been created, including *the Miscarriage and Abortion hotline (M+A hotline), a free hotline staffed by volunteer medical providers who provide medical advice to patients after they have begun a self-managed abortion or miscarriage.* Originally intended to be purely a medical consult service, many calls since the pandemic have also included emotional support. This hotline saw a marked increase in calls since the beginning of the COVID crisis.

## After an Abortion

Lorie (name changed) called the hotline because she needed to talk. She had been pregnant but was not ready to have a child. She was working and going to school and barely had enough money for her monthly expenses. She had gotten medication online because she could not afford the cost at her local clinic. She was too afraid to ask her family or friends because she was not sure what they would think. Two weeks ago, she had taken the medications, and the abortion had no complications. However, she was still feeling sad. “I feel so alone. I always tell my best friend everything, and I just can’t.” Reflecting on that isolation, we talked about why she felt she could not share with her usual support system. She identified some as having religious convictions that she felt would keep them from supporting her. Others, she just assumed, would judge her based on what feels like a societal norm of disapproval and the sense that it was “her” mistake.

*As a medical procedure, abortion is not a risk factor for developing mental health disorders* (Steinberg, 2014). After an abortion, support may or may not be required. Some patients will feel relief at the completion of a desired or necessary procedure. Others may feel a loss. This is typically magnified in patients who have abortions due to complications of a desired pregnancy. Isolation may occur due to perceived external shame, internalized stigma, or judgment from a patient’s community. This is particularly challenging as it may prevent patients from relying on their typical support systems. Here, COVID increased feelings of isolation as it diminished in-person contact among community members. Processing the source of shame, *the impact on one’s spiritual health*, and building on one’s conviction for why the abortion was right for them, can help to minimize the self-flagellation. Normalizing the fact that a patient is part of a common humanity with one in four women having an abortion in their life may also help patients to feel more connected to a community (Jones, 2017). Online sources, such as Exhaleprovoice.org, can help these patients access community and counselors experienced in this support.

## Conclusion

When I asked Stephanie about her tears, she explained that she often cried when frustrated. She had done everything she could to get the abortion done early in her pregnancy but her local clinic had closed temporarily due to COVID and it forced her to delay her care. She was able to complete her procedure without any complications. She left the clinic and drove home with dry eyes.

A patient’s journey through the decision to get an abortion, completing the abortion, and processing their experience is unique to every patient. Support requires holding a mirror up to patients to help them reflect back on their convictions. Once a decision is made, it is helpful to alleviate anxieties that may arise around the procedure itself. After completion, patients may not need any support at all. Others may need to address feelings of shame and isolation. Whether it is during a pandemic or not, it is important for there to be mental and spiritual support for patients accessing reproductive healthcare.
